# Practical Notes

**Published:** 1891-04

**Authors:** 


### Practical Notes.

FOR SUB ACUTE OR CHRONIC DIARRHŒA.—

℞ Acacia pulv., ʒ js.
Creosoti (beech wood), gtt xxxij.
Aquae purae, f ℥ iv.

Ft. Emulsio et. Sig. A teaspoonful in water (for adults) every 4 to 6 hours.

FOR PAINS OF DYSMENORRHŒA AND NERVOUS HEADACHE.—

℞ Morphine sulph., gr. j.
Aquae camphorae, f ℥ iijss.
Ether chloric, f ℥ ss.

Solve et. Sig. A tablespoonful in water occasionally.

—*Dr. A. W. Griggs.*

### DR. THOMAS S. POWELL'S ADDRESS TO THE GRADUATING CLASS SOUTHERN MEDICAL COLLEGE.

Gentlemen of the Graduating Class:

It gives me much pleasure to feel that by zealous application to the study of your chosen profession, you have honorably won your diplomas in the Southern Medical College.

In accordance with the laws of Georgia, you are now Doctors of Medicine, and in the name of the Trustees and Faculty I congratulate you, and give you the benediction of your Alma Mater.

I fear this name, which is so frequently and appropriately used in like occasions, is not often given with the significance that the term implies. The office of "cherishing mother," in a material and immaterial sense, is certainly one of much importance and exalted character.

A mother who cherishes her offspring in the true sense of the word, endeavors to develop its moral, mental and physical capacities to the utmost and noblest evolution. Over it her watchful care never ceases; her anxiety for its welfare never abates, and her tender affection enfolds it in a mantle of love and charity that no hands but its own ungrateful ones can tear away.

To such a parent is due peculiar duty and fidelity, and he who is her worthy son, is glad to give her lasting and loving allegiance. I believe, gentlemen, you will be faithful to your Alma Mater, and instead of ever bringing one blush of shame to the cheek, you will endeavor to make her heart throb with pride and gladness as she watches your future career, be it long or short.

Scarcely a generation ago, corruption in business circles, in professions, trade, society and morals was the exception instead of the rule. In every department of industry and science in the moral and the material world, there never has been a period when that impressive question, "Watchman, what of the night?" needed so much to be asked with earnestness and anxiety.

Now it is *vice versa*, and the slime of foul doing and foul dealing has inoculated with its virus the very heart of humanity.

There is so little confidence in the integrity of men, the world must see the best, the truest and noblest among them pass through long years of severe crucial tests—go down into a very Gehenna of fierce trials and temptation, and come out without the smoke of fire upon their garments before it will give them even the private recognition the grandeur of their character so richly deserves.

Yet from the very fact that exalted nobility of character is now so rare, there has not been a time in our memory when it could stand so conspicuous before men, and blaze like a star of the first magnitude amid the moral gloom that threatens to engulf individuals, society and the nation itself.

In your career as men and as physicians, my young friends, let that beacon light lead you into the luminous path beyond, and when you hear ringing through "the brooding darkness around you" that thrilling cry, "Watchman, what of the night?" I would have you be able to answer for yourselves, "Well! all's well!" We are on the watchtower of medical science, and earnestly striving to exalt our noble profession to lofty heights of character, and by its healing power and skill, wrest suffering humanity from the clutches of disease and premature death.

It is a shame that in a Christian country men should ever say what we so often hear on every side, "It does not pay to do right," meaning it does not pay in money—this god of the world's cringing idolatry ; it does not pay in position and success that can be bought for so many dollars and cents, like the commonest wares in a huckster's market.

But I hold that an honorable, upright career does pay. Though sordid motives never influence the ambition of the true physician, his ministrations are often rewarded in a highly pecuniary sense, and in being faithful to the pure, elevated nature of his profession, it always pays in something better than silver and gold—in winning the love, respect and reverence of his fellow men, and stainless, well-deserved honors, sooner or later. Wrong often succeeds for a time, but truth triumphs at the last, and while the defeated hosts of evil lie howling in despair and darkness, she beats the ambient air with her snow-white wings, and soars singing into eternal day.

Suffer me then, gentlemen, to entreat you, in these my parting words, to consider the importance of learning your whole duty, and discharging it faithfully. Become as nearly as possible perfect in your profession. Learn prudence and self-control. Have decision and independence of character. Aspire and strive to be men of the noblest and highest type. If you would compete with the world at large in its march of improvement, if you would mount the dizzy height of a laudable ambition, and unfold the hidden mysteries of the profession, you must study—constant and unwearied study can alone bring you to so welcome and happy a consummation of your desires. Remember, my young brethren, to improve a man is to liberalize and enlarge him in thought, feeling and purpose; cultivate his heart with moral and religious principles; above selfishness and with great intellectual culture, man triumphs over the universe—he rules all nature. Educated, independent mind knows no limit. It is halted only by the line that separates the soul of man from his Creator. It surveys the past, penetrates the future, and foretells what is to come; it ranges amongst the heavenly bodies and explores the recesses of ocean and the earth. Nothing on earth or in heaven eludes its search, or is too vast for its comprehension. My young brethren, the whole field of nature is yours; all the physical sciences are embraced in the science of medicine—the noblest science cultivated by human intelligence. But, gentlemen, remember, you may have the mind of a Watts or a Newton, and may be able to subdue the elements; you might have the power of Demosthenes, the eloquence of Cicero, the judgment of a Pitt, a Mirabeau or a Grattan, without an accurate knowledge of the human organization, and all the other branches which it has been our duty and pleasure to teach you in the Southern Medical College, the practice of medicine will be a mere pretension. To be a physician in the true sense of the word; to be able to control the most mysterious of the forces of nature; read the mysteries of man's interior formation; to be able to trace and detect disease in its most secret lurking places, you must continue to study, observe, reason and think. Study, constant and unwearied study, can alone bring you to so welcome and happy a consummation of your desires.

To accomplish all this, you must be a conscientious, honorable man.

The heart, as well as the intellect, must be highly cultivated. Let me then entreat you to strive to improve both.

In the very beginning of your professional life, I would have you faithfully observe the ethics and rules of the profession; but remember that the truly great physician not only labors continually to reach the acme of medical science so as to prevent as well as relieve physical ills, but he has the heart of gold that goes out in compassion and brotherly love and sympathy ready to minister without partiality or prejudice to all who need his services—the rich and the poor, the learned and the ignorant, the great and the low.

From the very nature of medical ministry, the life of a true physician is one of self-sacrifice. His mission is to serve, not to be served. Unless a man is willing to give freely of himself wherever his ministrations are needed, I would not advise him to enter the profession of medicine. Therefore, if you would be the physician who is loved and honored in his glorious life of good deeds and good will to men, at the beginning bring your talents, your hopes and aspirations, and lay them upon the altar of duty. Let no love of ease, self-indulgence and sordid ambition entice you into their deceptive snares, and let your private and professional life bring nought but good and richest blessings to the world.

In bidding you farewell, I would thank you for the courtesy and kindness shown to your instructors, and the assiduous attention given to your studies. May the benediction of heaven go with you to your homes, and rest upon you in all your life's career.

